# Metabarcoding by capture using a single COI probe (MCSP) to identify and quantify fish species in ichthyoplankton swarms

**DOI:** 10.1371/journal.pone.0202976

**Published:** 2018-09-12

**Authors:** C. Mariac, Y. Vigouroux, F. Duponchelle, C García-Dávila, J. Nunez, E. Desmarais, J.F. Renno

**Affiliations:** 1 Institut de Recherche pour le Développement, Université de Montpellier, Unité Mixte de Recherche Diversité Adaptation et Développement des Plantes (UMR DIADE), Montpellier, France; 2 Laboratoire Mixte International—Evolution et Domestication de l’Ichtyofaune Amazonienne (LMI—EDIA), IIAP—UAGRM—IRD, UMR BOREA, Paris, France; 3 Institut de Recherche pour le Développement, Unité Mixte de Recherche Biologie des Organismes et Ecosystèmes Aquatiques (UMR BOREA), MNHN—CNRS-7208—UPMC—UCBN—IRD-207, Montpellier, France; 4 Instituto de Investigaciones de la Amazonía Peruana (IIAP), Laboratorio de Biología y Genética Molecular (LBGM), Iquitos, Perú; 5 Institut des Sciences de l’Évolution (UMR ISEM), Université Montpellier—CNRS—IRD—EPHE, Place Eugène Bataillon—France; University of Guelph, CANADA

## Abstract

The ability to determine the composition and relative frequencies of fish species in large ichthyoplankton swarms could have extremely important ecological applications However, this task is currently hampered by methodological limitations. We proposed a new method for Amazonian species based on hybridization capture of the COI gene DNA from a distant species (*Danio rerio*), absent from our study area (the Amazon basin). The COI sequence of this species is approximately equidistant from all COI of Amazonian species available. By using this sequence as probe we successfully facilitated the simultaneous identification of fish larvae belonging to the order Siluriformes and to the Characiformes represented in our ichthyoplankton samples. Species relative frequencies, estimated by the number of reads, showed almost perfect correlations with true frequencies estimated by a Sanger approach, allowing the development of a quantitative approach. We also proposed a further improvement to a previous protocol, which enables lowering the sequencing effort by 40 times. This new Metabarcoding by Capture using a Single Probe (MCSP) methodology could have important implications for ecology, fisheries management and conservation in fish biodiversity hotspots worldwide. Our approach could easily be extended to other plant and animal taxa.

## Introduction

Currently nearly 35,000 fish species have been described, and this number is regularly increasing with ~400 new descriptions annually [1]. In fish biodiversity hotspots, such as coral reefs and the Amazon basin, the large majority of fish species have larvae that intermix in multi-specific ichthyoplankton swarms. Being able to determine the precise composition and relative contributions of species in these ichthyoplankton swarms would have extremely important ecological applications, such as biodiversity evaluations, identification of the locations and seasons of species’ breeding, and assessment of the relative contribution of particular reefs or tributaries to species recruitment. This information is pivotal for designing sustainable fisheries management practices and conservation strategies. Unfortunately, access to this crucial information is usually limited by the lack of appropriate tools. Precise specific identification is often impossible using morphological approaches, particularly for early development stages, also, while barcoding solves the identification problems, individual sequencing of each larva is tedious and expensive and thus becomes time- and cost-ineffective when large numbers of larvae are involved. The metabarcoding approach might provide an interesting solution for the massive and rapid identification of species in bulk samples. Until recently, such metabarcoding approaches used PCR based methods [2–9]. However, a PCR based approach leads to non-exhaustive species identification and does not always allow accurate species quantification [2–9]. These limitations are directly related to the difficulty of designing universal primers on the COI barcode because mutations in different sets of species in the primer site lead to non or poor amplification [10–12]. This bias leads to an underestimation of the specific diversity and makes it more difficult to obtain reliable quantitative data [13]. To overcome PCR biases, different PCR-free methods have been proposed, such as shotgun sequencing [14], [15] or mitochondria enrichment [16], [17]. However, these approaches remain costly because the low percentage of COI barcodes sequenced requires high sequencing depth. Hybridization is less affected by divergence and consequently approaches using this technique may allow better quantification of species composition. Although metabarcoding by capture has been suggested as an alternative to the PCR approach since 2012 [18], very few studies have been carried out [19–22]. One of the key elements of the capture approach is the probe used to capture the COI barcodes. Usually, biotinylated probes capable of hybridizing with the homologous sequences present in the library preparation are used for target enrichment [2–9].

We previously successfully used enrichment by capture to develop a method allowing quantification of siluriform species in Amazonian ichthyoplankton samples, using four different probes distributed in the main clades of the phylogeny of Amazonian siluriform species [21]. Yet, siluriform species represent only ~20% of the Amazonian fish diversity. Extending this method to the other fish orders (Characiformes, Perciformes, Gymnotiformes, etc), would require so many probes to cover all clades that it would be technically difficult. Here, we propose a new approach using an almost universal probe able to capture all main fish orders of the Amazon basin. We validated the accuracy of this new method and obtained a very good estimate of species frequencies for ichthyoplankton swarms from the Amazon basin.

## Materials and methods

### Selection and preparation of the probe

Our approach relies on a single COI probe (MCSP: Metabacording by Capture with a Single Probe). We chose a COI probe (hereafter named single-probe) from a species (*Danio rerio*) belonging to an order (Cypriniformes) absent from our study area, the Amazon basin.

The single-probe was developed by PCR amplification of the CO subunit 1. PCR was performed with 80 ng D. *rerio* DNA, 0.3mM of forward and reverse [5']-Biotin-TEG primers [23] (FishF = TCA-ACC-AAC-CAC-AAA-GAC-ATT-GGC-AC, FishR = TAG-ACT-TCT-GGG-TGG-CCA-AAG-AAT-CA), 12.5 μl HiFi PCR kit (KR0369, KAPA Biosystem), 9 μl H2O. PCR was run with the following cycling protocol: 95–3’, 98–80”, 52–40”, 72–1’- 35 cycles [24].The PCR product (682 bp) was purified with 1X ampure XP and then quantified.

In this study, we will compare the result from this new approach to a previous approach using four probes designed for siluriforms [21]. In the following text, we will use the term single-probe for the COI from Danio and the term siluriform probes for the four probes previously developed [21].

### Samples used for validation

To validate the effectiveness of our protocol, we used two batches of larvae, one from the Marañon River and one from the Napo River. These two batches were randomly divided into two subsets. The first subset was used to perform individual DNA extractions and were then Sanger sequenced [21]. These individual DNAs (270 individuals from the Marañon River and 102 from the Napo River) were pooled in equimolar quantities to constitute mock samples (Mar-Mock and Nap-Mock). These mixes of DNA simulate swarms, from which we know the exact taxonomic composition.

The second subsets (named Mar-bulk and Nap-bulk) were processed as true swarm samples. All individuals from each of the two rivers were extracted in a single DNA extraction. The Napo sample had 250 larvae, and the Marañon sample had 373 larvae. We expected the species compositions of these two subsets to be close to the two mock samples. These two last subsets allow us to assess variability associated with bulk DNA extraction of larvae.

### Libraries preparation and enrichment

All steps and conditions of library preparations (DNA shearing, DNA end repair, primer ligation, Bst Polymerase treatment and real time PCR) follow already published protocols [21], [25]. For each DNA library, each capture was performed on 200 ng of DNA with 200 ng of biotynilated probe. In solution hybridisation was carried out in 40 μl in a final concentration buffer of 6 X SSC, 0.05% SDS, 0.3 ng/μl BSA, 0.12 ng/μl salmon sperm DNA, and 2.75 μM of each oligonucleotide blocking OB-P5: AGATCGGAAGAGCGTCGTGTAGGGAAAGIIIIII and OB-P7: AGATCGGAAGAGCGGTTCAGCAGGAATGCCGAG). After denaturation of the DNA for 5 minutes at 98°C, hybridization was performed at 55°C for 4h30 and 16h for the first and second round of capture, respectively. After hybridization, biotinylated probes-target complexes were bound by adding 40 μl of binding buffer (10mM Tris-HCl, 1mM EDTA, 2 M NaCl) containing 0.2 mg of streptavidin-coated magnetic beads (Dynabeads® MyOne™ C1, Invitrogen™) for 30 minutes at 55°C. Beads were then washed with 150 μl of 1X SSC, 0.1% SDS for 5 minutes at 55°C followed by three 5-minutes washes at 55°C with 0.1X SSC, 0.1% SDS and a final wash with 0.2X SSC for 5 minutes at room temperature. Beads were then resuspended in 15 μl H2O and single-stranded DNAs eluted after 5 minutes at 98°C. DNA was then amplified with primers targeting the P5 and P7 regions of Illumina TruSeq® adapters for 17 cycles. Libraries were paired-end sequencing using MiSeq v2 reagents and 2 × 150 bp. Sequencing was carried out at the CIRAD facilities (Montpellier, France).

### Construction of the COI database

A local database was built with 106,494 Actinopterygii COI sequences extracted from Genebank on February 21^st^ 2017. Details of the command line used for extraction and of sequences manually added or removed are listed in supporting information (S1 Text). The final database represents 2,837 genera corresponding to 7,213 species from which 7,068 are named (S2 Text). The database contains 444 COI barcodes from Amazonian fish species. We aligned these sequences using ClustalW2 [26] and calculated pairwise genetic distances between these 444 sequences using the MEGA 7 program [27].

### Data cleaning and taxonomic assignation

Demultiplexing based on the 6-bp internal index was performed using the PYTHON script DEMULADAPT (https://github.com/Maillol/demultadapt). Adapters were removed using CUTADAPT 1.2.1 [28]. Reads with a mean quality lower than 30 were discarded using a freely available PERL script (https://github.com/SouthGreenPlatform/arcad-hts/blob/master/scripts/arcad_hts_2_Filter_Fastq_On_Mean_Quality.pl). The Sanger and NGS sequences were aligned with MALT version 0.3.8 [29] against our COI database (command line in S1 Text). The sequences generated by Maggia et al (2017) and those obtained in this article were processed using the same workflow. Taxonomic assignation was performed with MEGAN software version 6.8.5 [30] using the naïve LCA method. The number of reads with similarity or without similarity to our COI database was evaluated. Reads mapping COI were assigned to a species or a higher taxonomic level when the read had a minimum alignment score value of 230 [30] and 99% identity with a reference sequence in the database. Reads with similarity to COI were consequently considered assigned or not assigned. Reads not assigned represent reads with some similarity (partial sequence, …) to COI in the database but that did not pass our strict assignation filters. Finally, reads without hit correspond to reads that match no COI sequences in the database. The percentage of reads with some similarity to COI was used as a measure of the capture efficiency [(number of reads with similarity to COI) / total number of reads]. A Shotgun Genomic library was used to calculate the percentage of reads mapping the COI database before enrichment. This capture efficiency is measured as the X-fold enrichment, i.e. the ratio of the percentage of reads from the enriched library mapped to the reference database compared to the percentage of reads from the unenriched (genomic) library mapped to the reference database.

### Species composition and relative frequencies

To accept the presence of a species, we fixed a minimum number of reads per species. This threshold was determined for each mock sample by comparing real species composition (established with Sanger sequencing of individual larvae) and the species frequencies estimated through NGS data. We calculated the true positive rate (sensitivity) and true negative rate (specificity) using the R package ROCR 1.0–7 [31], [32]. We then used the method of maximum sum of sensitivity and specificity (maxSSS) to determine the optimal threshold [31], [33]. This approach calculated the threshold corresponding to the highest total value of sensitivity plus specificity. A species with reads count below this threshold was considered absent. Threshold values were calculated for the mock libraries: 2 sampling sites (Marañon or Napo) and 2 types of probes (siluriform probes or the single-probe). Once this threshold had been applied to the NGS data, we calculated the species composition of each sample. We also calculated the relative species frequencies for each sample.

## Results

### Single probe design and properties

**Fig 1:**
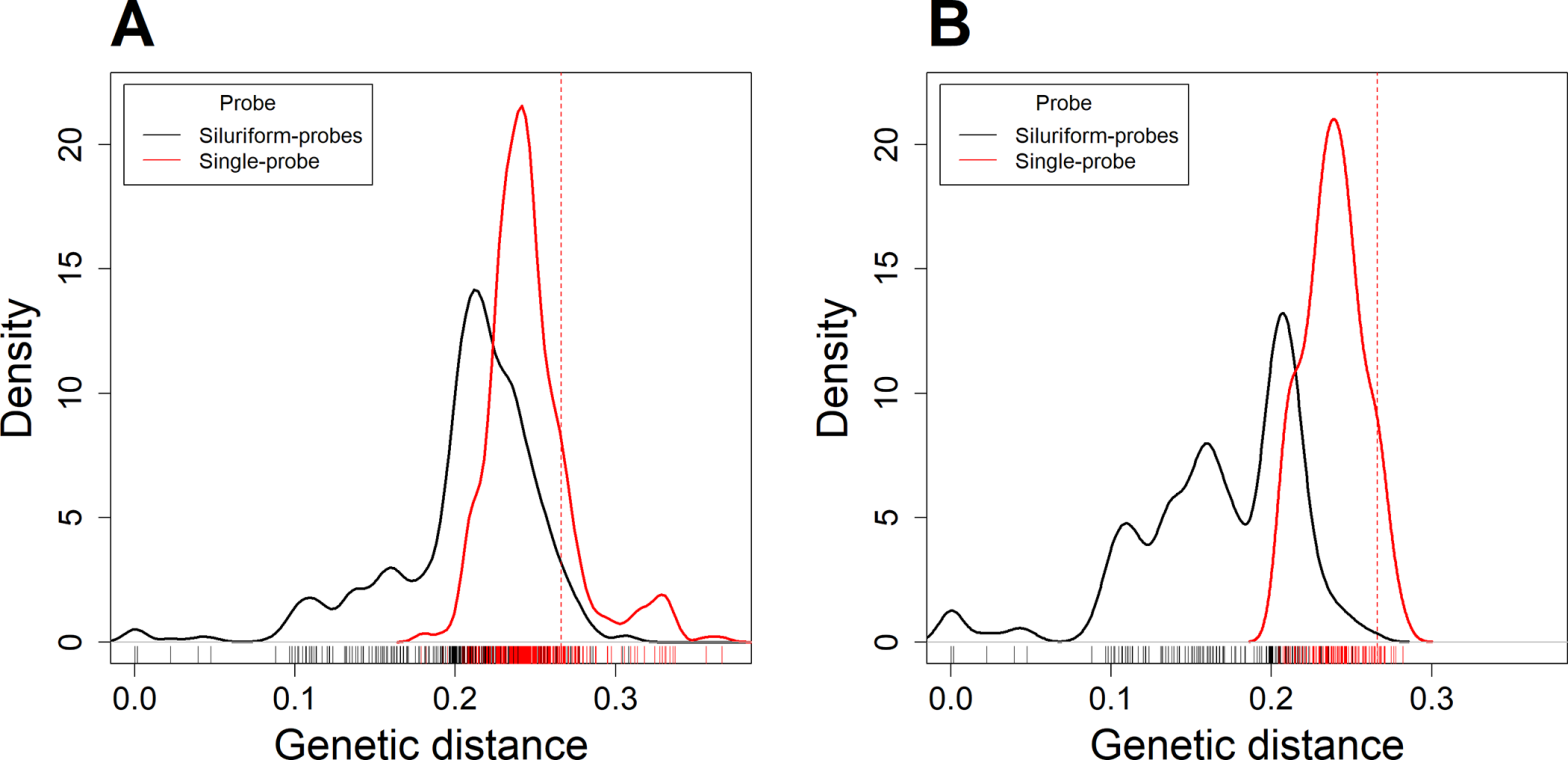
Density distribution of genetic distance. A. Density distribution of genetic distances (Juke Cantor 1969) between the COI barcode sequences of 444 Amazonian fish species and the single-probe (red) and siluriform probes (black line). The dashed red bar line corresponds to the most distant species in our experiment with a genetic distance of 0.26. B. Density distribution of genetic distance for the 164 siluriform species only.

The single probe we designed presents a mean divergence of 24.6% and a Coefficient of Variation of 10.9%, (Fig 1A and S1 Table) to the 444 Amazonian fish species in our COI database. The probe is nearly genetically equidistant to these 444 species belonging to 15 different fish orders [34], [35]. The four siluriform probes we previously used [21] had slightly lower divergences (20.6%) but the Coefficient of Variation was twice as high (22.5%). If we focus on the siluriform species only, the Coefficient of Variation for the single-probe and siluriform probes are 7.5 and 30.3%, respectively (Fig 1B).

### NGS sequencing and capture efficiency

To assess the effectiveness of our approach, we obtained 2.4M reads after capture with the single probe (see Table 1 for details per library). In order to allow an easy comparison with previous datasets, we reanalysed all of them using the workflow developed here (~ 4.7M sequences generated using capture with the siluriform probes, S2 Table).

**Table 1:** Number of reads and assignation rate for libraries enriched with single probe. The total number of reads and assignation results against COI database is reported at both sites: Marañon and Napo rivers and for both samples: Mock and Bulk. For each sample the number of larvae is given. “Reads with hit” indicates the number of reads mapped to the COI database but not taxonomically assigned because the threshold filters (min bit-score 230 and identity 99%) were not met. “Reads assigned” accounts for the number of reads where taxonomic assignation was successful. Capture efficiency was estimated through the X-fold enrichment and calculated as the ratio of the percentage of reads with hit between an enriched and an unenriched library (see text for details).

Site	Sample	Number of larvae	Number of NGS reads	Reads with hit (%)	Reads assigned (%)	Capture efficiency
Napo	Mock	102	969,742	673,054 (69.4)	272,347 (28.1)	5,904
	Bulk	250	161,658	119,070 (73.7)	49,063 (30.3)	6,265
Marañon	Mock	270	894,106	600,112 (67.1)	240,679 (26.9)	5,709
	Bulk	373	428,880	338,362 (78.9)	130,557 (30.4)	6,711

The capture with the single-probe showed a percentage of COI reads after enrichment varying from 67.1% to 78.9%. The number of COI reads in the unenriched library was only 0.012%. Therefore the new method improved enrichment ~ 6,000 times compared to an unenriched library.

The mean percentage of reads that reached a hit in our COI database was 70.5% (Table 1). As we applied stringent thresholds for both percent identity (99%) and bit-score value (230), only 28.2% of the reads were assigned to a species or a higher taxonomic level. With the very stringent thresholds used, the near-full length of the reads (~140 bp) must be aligned to a barcode reference to be assigned. Consequently partially mapped sequences presenting a COI hit were not kept for species identification. We expected reads in flanking regions of the COI target upstream and downstream of the probe [36], [37] to be excluded during the assignation step. A total of 29.5% reads yielded no hit in the COI database, a value consistent with other studies using enrichment by capture on mitochondria and performed with commercial kits[38–40]**.** A blastn alignment on a subset of these reads (~ 290,000) on the nucleotidic collection (nt) NCBI database (Jan 3, 2016) showed that most remained not assigned (91.7%), about 7% were assigned to fish (Actinopteri) and 1% to other eukaryotes. Only a few of these reads were assigned to bacteria: 0.02%. Altogether either hitting on COI fish database or on NCBI database, 72.7% of reads proved to be of fish origin.

### Sensitivity

We first wanted to measure our ability to identify species (sensitivity), by comparing the NGS capture results to the true composition established by the individual Sanger sequencing of the larvae (S3 Table). Of the 372 Sanger sequenced larvae, 367 larvae were identified at the species level, three at the genus level, and only one larva could not be assigned (S4 Table). Taxonomic diversity of the mock samples was distributed across 3 orders, 10 families, 22 genera and 29 species (11 from the Napo and 25 from the Marañon, with 7 common species between the two rivers). All species present in the mock samples were successfully identified with our new protocol (sensitivity = 1.0), (S5 Table). When we reanalysed the data from our previous study [21] using the siluriform probes, sensitivity was only 0.69 with 25 species identified and 11 not identified of which 7 belonged to the order Characiformes. These false negatives corresponded to species present at low frequencies (mean = 1.36%, SE = 0.86).

The sensitivity of our approach based on 140bp fragments was very good to identify species. In order to explain this result, we performed an empirical simulation. We used the 444 different Amazonian species COI sequence from the database. We then simulated we had only partial sequences of these 444 species (i.e reads of 50bp, 100bp, 140bp, 250bp) or the full COI sequence. We then assigned these reads to the full COI database representing 7231 species (S6 Table). Initially we assessed the quality of the database just based on the full COI sequence length. With a perfect database we expect 100% of the COI reads to be assigned at the species level. We observed that 95.7% of the COI were assigned at the species level. The difference might be accounted for by species sharing similar COI sequences, and consequently the COI is attributed at a higher level (genus, family,…). This could occur between weakly divergent species or because of taxonomic problems in the database, such as a same species having two different names. Hence, 95.7% is the highest species assignment rate that could be expected for shorter fragments.

For a size of 140 bp, 83.5% of the reads were assigned at the species level. This means that, when assigned at the species level, a read has a single "best hit" across the 7230 species in our COI database. This rate reached 87.8% with 250 bp reads, but it remains fairly high even for very short fragments: 68.0% for 50 bp. This is an interesting result because even for short read from partially degraded DNA often found in environmental DNA experiment, we will still have an appreciable assignation rate at the species level. Based on this simulation, the true positive rate was very high (93.7%) for 140 bp fragments and only three false positives out of the 444 species were observed. True positive rate ranged from 93.0% for 50 bp up to 93.9% for 250 bp. The number of false positives was 20 for 50bp and decreased to 2 for 250 bp. To explain this high specificity, we aligned the 444 Amazonian COI sequences and counted the number of SNPs differentiating the species (by pairwise comparison). We found an average of one polymorphic position (segregating site) every 7.2 bp. Hence, for 50 bp we expect an average of 7.9 segregating sites between two species.

In our empirical data, we found 88.4% of reads assigned at the species level. Using reads of 250 bp instead of 140 bp would only increase the assignment rate at species level by only 4.4%. On the whole, our sequencing strategy using sequence around 150bp is adequate for the purpose of the study.

**Fig 2:**
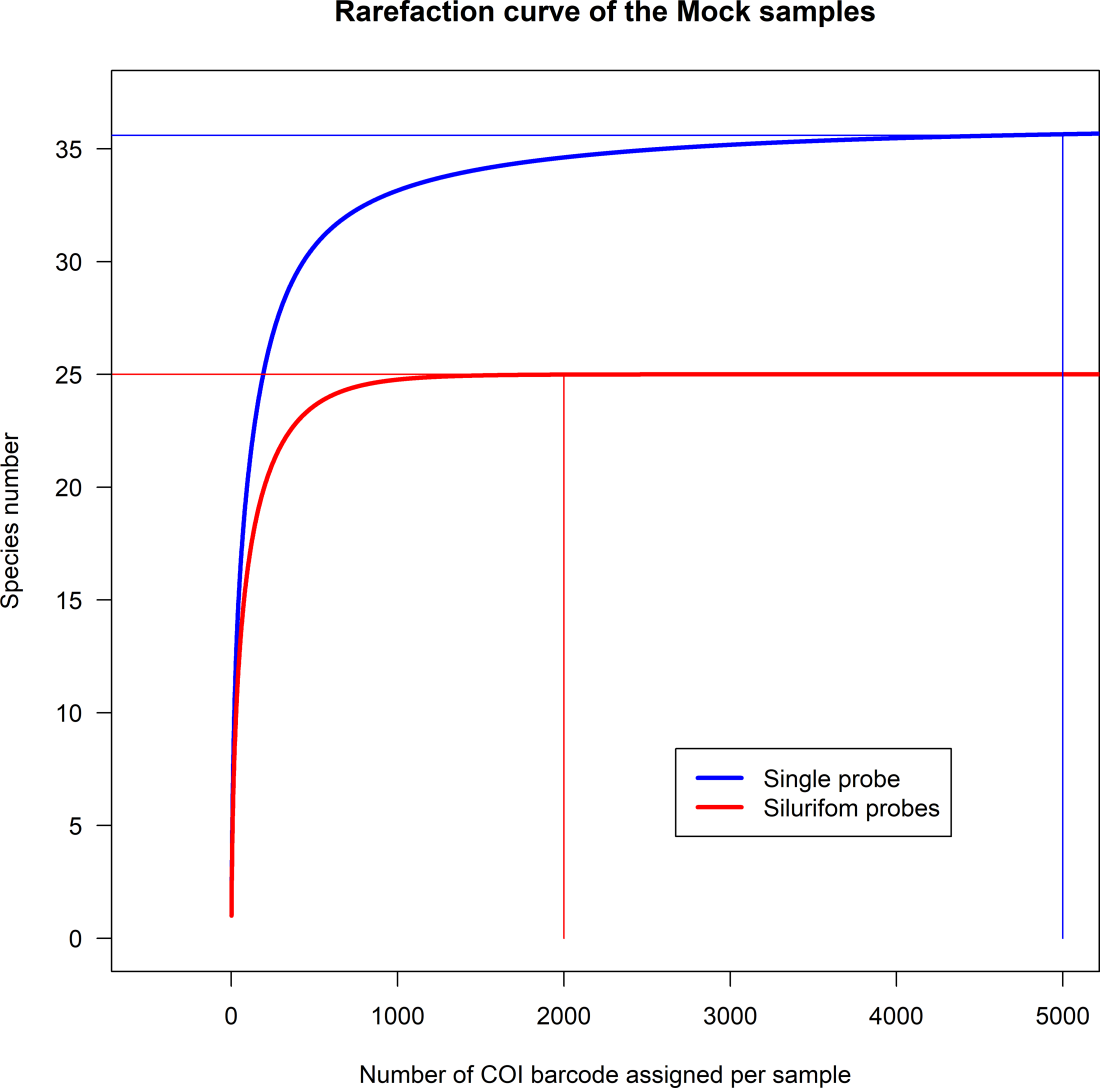
Rarefaction curves. The curves represent the number of species identified among the 36 species (Characiformes and Siluriformes) in two mocks as a function of the number of COI barcode assigned. With the siluriform probes (red line), at most 25 species are identified with 2,000 COI barcodes (asymptote). With the single-probe (blue line), 34 species are already identified with 2,000 COI barcodes and all 36 species are identified with 5,000 COI barcodes (asymptote).

Owing to a more intensive sequencing effort and enrichment efficiency, the number of COI reads assigned with the single-probe was 77 times higher than that obtained with the siluriform probes. We assessed if this sequencing efficiency could explain the effectiveness of the single-probe. To do so, we calculated rarefaction curves in order to estimate the minimal number of reads needed to identify all our species. Using the siluriform probes the asymptote was reached with 2,000 COI reads and allowed the identification of 25 species (Fig 2). In contrast, using the single-probe, 34 species were already identified with 2,000 COI barcodes. This demonstrates that the highest sensitivity obtained with the single-probe is not due to the difference in sequencing effort (Fig 2). In addition, the sensitivity of the single-probe is such that it was possible to identify species efficiently even when their frequencies were below 1% and their genetic distance to the probe as distant as 0.26 (*Leiarius marmoratus*).

### Specificity

The number of false positives in the mock sample enriched with the single-probe was five. All false positives (*Colossoma sp*. *KU 3081*, *Leporinus trifasciatus*, *Prochilodus lineatus*, *Prochilodus rubrotaeniatus*, *Semaprochilodus kneri*) excluding *Colossoma*, were close to congeneric species present in the mock and all had very low relative frequencies (mean = 0.42%, SE = 0.33). *A posteriori* alignment of *Colossoma macropomum* (the only species in the genus) barcodes sequences from our database indicated that *Colossoma* sp. KU 3081 (GenBank: FJ918909.1) had a too low identity (87.3%) to be considered as *Colossoma* and can therefore be considered taxonomically misidentified.

Analyses of genetic distance between *Piaractus mesopotamicus* and *P*. *brachypomus* showed that COI barcode alone is not enough to discriminate between these two species. However, as *P*. *mesopotamicus* is only distributed in the Parana-Paraguay basin, erroneous assignations to *Piaractus* and *P*. *mesopotamicus* can directly be attributed to *P*. *brachypomus*, the only species of this genus in the Amazon basin.

### Frequency estimation

**Fig 3:**
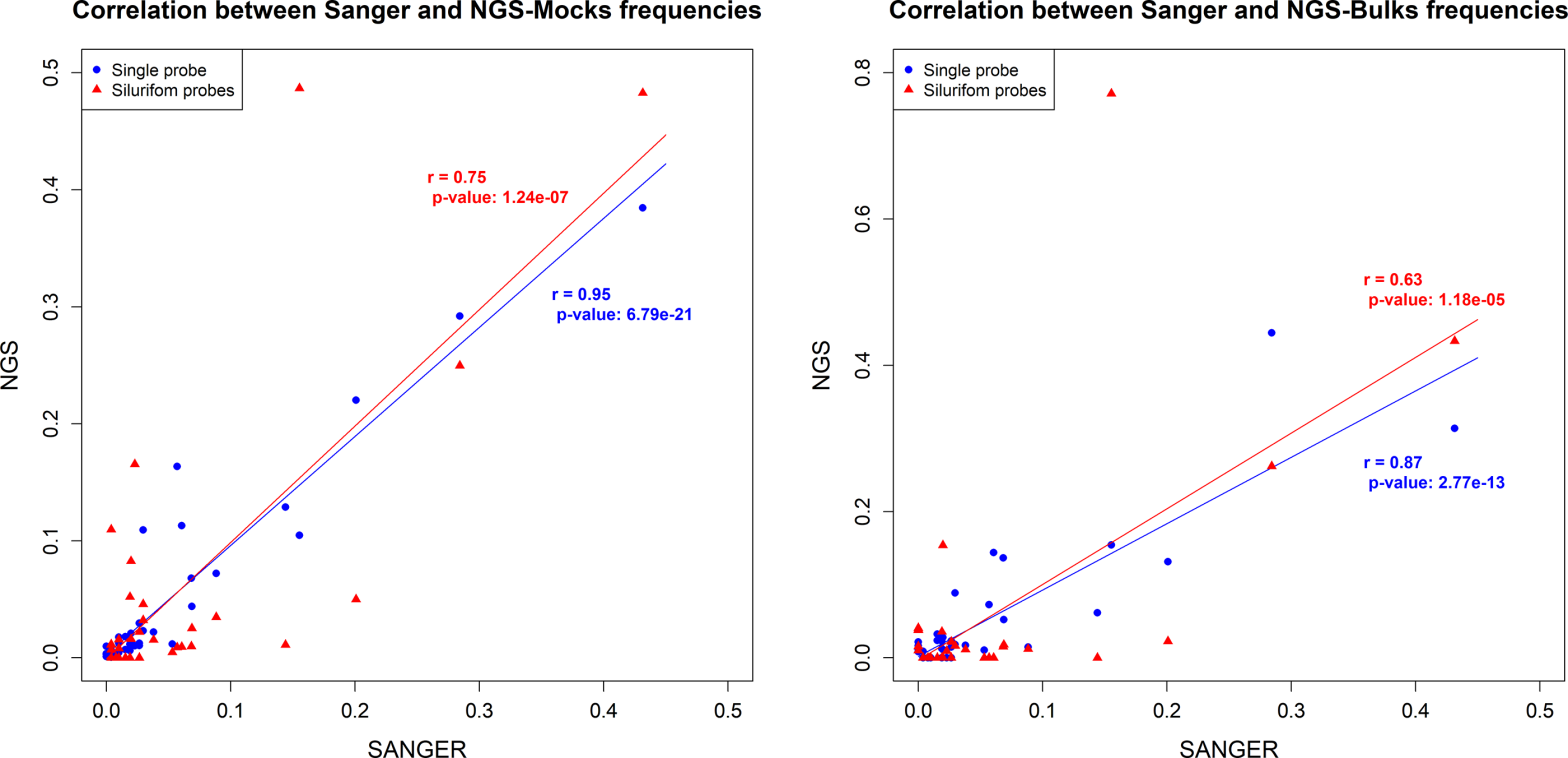
Correlation of frequencies. Correlations between Sanger frequencies of species (actual frequencies) and frequencies estimated from NGS libraries enriched with siluriform probes (blue diamond) or single-probe (red square) in mock (A) and bulk samples (B).

**Fig 4:**
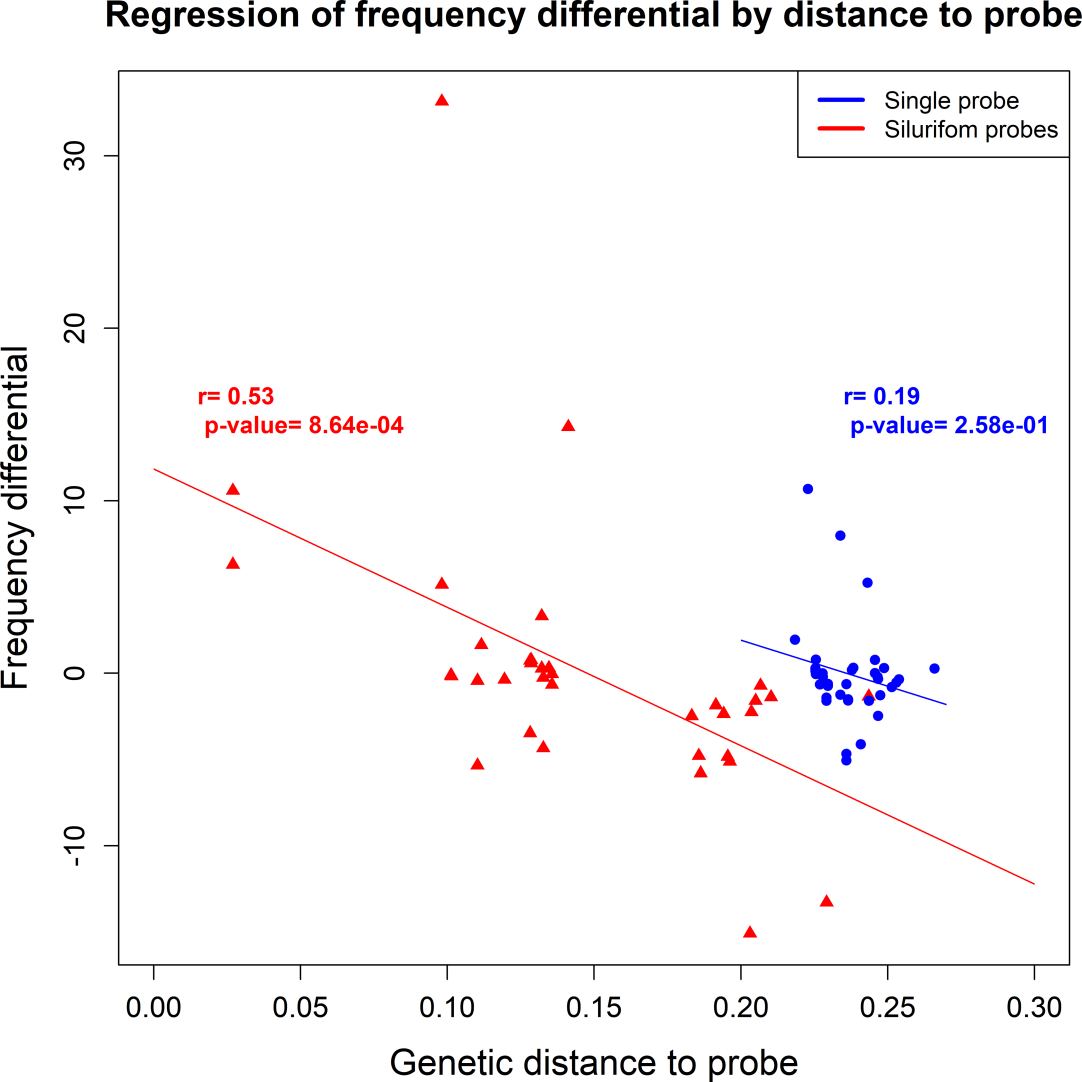
Influence of probe proximity on bias in the frequency estimation. For the siluriform probes, we found a significant correlation between the bias in the frequency estimation and the distance to the probes (red). We did not find such bias with the single-probes (blue).

The correlation of the species frequencies between Sanger and mock samples was very high: *r* = 0.95, n = 41, p < 0.001 (Fig 3A). Such strong correlation was found using the mock sample but also using DNA of larvae extracted in bulk (Fig 3B; *r* = 0.87, n = 40, p < 0.001). Moreover, there was no relationship between the genetic proximity of a given species to the probe and an enrichment bias (Fig 4; *r* = 0.19; p = 0.258).

Using the four siluriform probes led to a weaker correlation of species composition (mock: *r* = 0.75, n = 36, p < 0.001; bulk: *r* = 0.63, n = 41, p < 0.001) (Fig 3B) and bias of species/probes genetic distances (Fig 4; *r* = 0.53; p < 0.001).

## Discussion

We have proposed a new and effective approach for metabarcoding larvae in ichthyoplankton swarms. This new approach leads to 70.5% of reads mapped to the COI database, corresponding to a 6000-fold enrichment of COI sequence compared to native DNA. This represents a major gain compared to our previous protocol [21] where only 0.57% of the reads related to the COI barcode, with only a 137-fold enrichment. Consequently, this new protocol with a double capture of COI helps to reduce sequencing effort by 40 times.

The advantage of our approach is that it allows frequency estimation of species with high accuracy. We first evaluated this on samples made up of DNA from known species (mock samples), but also in “field-like” conditions (bulk samples). Bulk sample analyzes took into account the variability associated with extracting multiple whole individuals at the same time and variability in the size of larvae. The quantification remained very good on these bulk samples (r = 0.87).

The genetic similarity between the probe and the sequence to be captured can impact hybridization efficiency [40–42]. As a result, the species that have high and low sequence similarity to the probes are over- and underestimated respectively. This bias was observed in our case study when the four siluriform probes were used. The use of the simple distant probe significantly minimized this bias.

The mean divergence between the probe and all Amazonian species is 0.246. Even with this high divergence, the probe was highly effective at capturing all the different species in our samples. In this study, the mock samples were composed of 36 different species that covered the phylogenetic breadth of Amazonian fish species quite broadly, and represented the two main Amazonian fish orders. Characiformes and Siluriforms indeed represent over 73% of the diversity of South American freshwater fishes [43]. As the single-probe was proved efficient at capturing species at least as genetically distant as 0.26, we can assume that our method is likely to be effective for most Amazonian species. It is worth noting that, as we did not have species with higher genetic distances than 0.26, the single-probe might well be able to identify more distantly related species. But even considering that only species with a genetic distance ≤ 0.26 could be captured with the single-probe, this represents 84.7% (376 out of the 444) of the Amazonian fish species for which a COI barcode is available in public databases, including species belonging to most other orders such as Perciformes, Gymnotiformes or Osteoglossiformes (Fig 1).

It must be emphasized that our approach, like others [44], can lead to some false positives (species detected but actually absent). The species present in the sample but without references in the database can be assigned to a reference of a genetically close species, yet not the correct one. This is more likely to happen when many references are missing in the database. We have 444 Amazonian species in our COI database, which is a relatively low number compared to the 1,064 species currently described for the Peruvian Amazon alone [34]. We have therefore applied very stringent filters to limit these false positives. Databases of barcodes such as BOLD [45] are daily implemented thanks to the numerous studies carried out on aquatic biodiversity so we can hope that the incompleteness of these databases will be overcome.

Improving the referencing of all Amazonian species in the database is a pressing issue. As previously observed [46–48], having a representative and high quality reference database is a major factor to ensure reliable identification. The issue might be more pronounced for tropical fish species where diversity is very high and several species remain undescribed [49] We emphasize that the reference database must be updated and curated by regularly removing misidentified vouchers and poor quality barcode sequences.

There is an increasing interest [50] in using metabarcoding approaches on environmental DNA (eDNA). Our approach by capture, contrary to the PCR approach, might be of interest for dealing with partially or highly degraded DNA, which is frequently the case in environmental barcoding samples (eDNA). Capture for fragmented and low amounts of target is a protocol already used in ancient DNA [22], [38], [51], [52]. In such cases the enrichment is done on highly fragmented DNA. Also, as demonstrated in our study, 68% of the reads having a length of 50 bp were assigned to the species level, demonstrating the good taxonomic resolution of the COI barcodes even with very short fragments. It would be interesting to evaluate this capture enrichment approach on environmental samples. Our current protocol will have to be adapted to take the strong degradation of the eDNA into account and also that eDNA from macro-organisms in environmental water samples is at low concentration [53–56].

As PCR production of the probe is easy and inexpensive, it is possible to produce probes to capture other barcodes, such as 12S, 16S, 18S, ITS or MatK.

## Conclusion

By choosing a species in a taxonomic group absent from the study area to produce a single-probe equidistant from the potentially captured species, we significantly improved the estimation of species frequencies. This new strategy opens the way for quantitative studies of Amazonian fish recruitment using standardized ichthyoplankton samplings. In the mega-diverse Amazon basin, where the development of hydroelectric impoundments is increasing alarmingly [57–61], quantifying the relative importance of particular tributaries or sub-basins to the recruitment of commercially and/or ecologically important fish species will be a powerful tool to inform and guide decision-making. Access to fish recruitment will also have important implications for fisheries management and conservation. This MCSP approach could be successfully applied to metabarcoding of fish larvae in other mega diverse areas, such as coral reefs, and to a large array of animal and plant taxa. It also holds potential for eDNA studies.

## Supporting information

S1 Text Command lines and softwares used barcode. (R)

S2 Text COI reference database used in this study for taxonomic assignation. (FASTA)

S1 Table Genetic distance between COI probe and COI of 453 Amazonnian fish species. (XLSX)

S2 Table Number of reads and assignation rate for libraries enriched with Siluriform probes. (XLSX)

S3 Table ROC results and maxSSS values calculated for each library. (XLSX)

S4 Table List of Sanger sequences (Maggia et al. 2017) with additionnal taxonomic assignations performed in this study. (XLSX)

S5 Table Species frequencies calculated for each library. (XLSX)

S6 Table Assignation of hash reference barcodes. (XLSX)
